# Current Status of Core Competencies of Chinese Nurses in Burn Departments: A Latent Profile Analysis

**DOI:** 10.1155/2023/8839286

**Published:** 2023-04-30

**Authors:** Ping Feng, Jianling Hao, Yuan Wang, Xiaoying Lu, Yuanyuan Zha, Xinyu Li, Lin Zhou, Ning Li, Jianfang Zhang, Qin Zhou, Shujun Wang, Liyan Gu, Lingjuan Zhang

**Affiliations:** ^1^Burns Unit, Changhai Hospital Affiliated to Naval Medical University, Shanghai, China; ^2^General Surgery Department, Changhai Hospital Affiliated to Naval Medical University, Shanghai, China; ^3^Department of Nursing, Changhai Hospital Affiliated to Naval Medical University, Shanghai, China; ^4^Institute of Burn Research, State Key Laboratory of Trauma, Burn and Combined Injury, The First Affiliated Hospital of Army Medical University, Chongqing, China; ^5^Suzhou Hospital Affiliated to Nanjing Medical University, Nanjing, China; ^6^Burns Unit, Xijing Hospital Affiliated to Airforce Medical University, Xi'an, China; ^7^Burns Unit, Chinese Police Liberation Army General Hospital, Beijing, China; ^8^Department of Neurology, No. 905 Hospital of PLA Navy, Naval Medical University, Shanghai 200052, China; ^9^Education and Scientific Research Department of Clinical Nursing, Changhai Hospital Affiliated to Naval Medical University, Shanghai, China

## Abstract

**Aim:**

To investigate the current status of NBDs' core competencies through latent profile analysis, identify potential subgroups and their population characteristics, and analyze the influencing factors of different categories.

**Background:**

NBDs are essential in the treatment and rehabilitation of burn patients. However, the core competencies of Chinese NBDs are seldom reported.

**Methods:**

Our analyses were based on a cross-sectional and multicenter study of 267 Chinese NBDs. Latent profile analysis was employed to identify NBDs' core competence profiles using the NBD Core Competencies Self-rating Scale (NBD-CCSS). We then explored the characteristics among different profiles and determined socio-demographic variables associated with profile membership by conducting ANOVA, Chi-square test, and multinominal logistic regression analyses.

**Results:**

A 3-profile model provided the best fit. The three profiles were titled “skillful competencies” (Class 1, *n* = 77, 28.8%), “moderate competencies” (Class 2, *n* = 140, 52.4%), and “poor competencies” (Class 3, *n* = 50, 18.7%). Regression analysis suggested that professional title, years of employment, and BICU experience were influencing factors of NBDs' profile membership of core competencies. NBDs who were supervisor nurses or above (OR = 0.802, 95% CI: 0.009, 0.759), with more than 7 years of employment (OR = 0.091, 95% CI: 0.009, 0.906) and BICU experience (OR = 3.564, 95% CI: 1.423, 8.925) were more likely to fall into Class 1.

**Conclusions:**

Our findings could provide evidence for nursing administrators to develop training programs to enhance NBDs' core competencies. In particular, variables associated with profile membership determined in the study may facilitate more tailored training strategies.

## 1. Background

Burns can be caused by various factors such as fire, chemicals, or electricity, and they can lead to a significant financial burden on families and society [1]. Severe burns in China have an overall mortality rate ranging from 9.79% to 14.21% [2], highlighting the severity of this public health problem, particularly in developing countries where access to medical care is often limited [1]. Burn injuries can result in high rates of morbidity and disability, severely impacting the quality of life and psychological well-being of burn victims [3]. Treatment of burns can be costly and often requires long-term care [4]. Given that nurses are the primary healthcare providers who interact with patients frequently during their hospital stay, it is imperative for nurses in burn departments (NBDs) to be equipped with advanced professional knowledge and specialized skills to provide high-quality care.

As is known, the definitions, mechanisms, and development of nurses' competencies have been explored in-depth in western countries [5–7]. Similarly, studies on specialized nurses in intensive care, operating rooms, emergency medicine, oncology, wound care, and intravenous therapy have been conducted frequently in China [8]. Relevant studies have also expanded to address vulnerable populations (elderly, infants, children, and pregnant women), chronic diseases (e.g., diabetes, chronic cardiovascular, and cerebrovascular diseases), and the improvement of quality of life (e.g., nutrition and rehabilitation) [9–12]. Moreover, previous literature suggests that practical assessments of nurses' critical thinking and clinical reasoning competencies in real-world contexts could improve the quality of nurses' work [13]. Despite the progress made in these areas, there is still a lack of research on the current status and training of NBDs' core competencies in China. Furthermore, the scope and standards of NBDs' core competencies have not been standardized yet.

To date, registered nurses are expected to possess core competencies that encompass knowledge, skills, and attitudes to provide high-quality and safe care to patients. The Burn Nurse Competency Initiative (BNCI) developed 45 burn nurse practice competency statements through a multistaged consensus-building method by the American Burn Association (ABA) [14]. In light of their contributions, Chinese researchers have also developed instruments to assess NBDs' competencies [15], while the “National Expert Consensus on Professional Standards for Chinese Nurses of Burn Department” was constructed with definitions, operating contexts, occupational/skill requirements, training, assessment, and certification. The Chinese Expert Consensus categorizes NBDs into junior, intermediate, and senior groups based on their professional level [16]. However, it is important to note that the Chinese expert consensus was not specifically for burn-specific principles but established for job qualifications.

As noted earlier, NBDs are crucial in burn prevention, emergency care, and continuity of care in developing countries [17–19]. Despite their importance, newly hired NBDs are often placed directly in burn wards or BICUs without specialized training. Instead, their training focuses on fundamental knowledge, leaving them lacking in the specific competencies necessary for burn care. Developing specialized skills in clinical settings requires repeated practice, which many novice NBDs are not receiving. At present, the competency profile of NBDs is inconsistent across China, as this field of work is still in its early stages. Thus, a comprehensive assessment of NBD competencies is necessary to ensure they are fully equipped for their vital roles.

To better understand NBDs' core competencies, we have previously reported their overall competencies and factors affecting them [20]. In this research, we tried to utilize latent profile analysis (LPA) to help nursing administrators and policymakers tailor training programs. LPA allows researchers to identify subgroups of individuals based on shared attributes, enabling them to determine potentially diverse patterns of NBDs' competencies [21]. This person-centered approach has been used in previous nursing studies to identify subgroups related to workload, mental health, healthcare beliefs, or behavior, revealing associations with demographic characteristic [22–24]. By leveraging LPA findings, targeted actions can be taken to achieve favorable outcomes.

### 1.1. Study Aims

The purpose of this study was to investigate the current status of NBDs' core competencies through latent profile analysis, identify potential subgroups and their population characteristics, analyze the influencing factors of different categories, and provide a basis for developing targeted training programs.

## 2. Materials and Methods

### 2.1. Study Design

A cross-sectional study utilizing purposive sampling was carried out in 12 tertiary hospitals in China from March to June 2020. All study procedures followed the Karolinska Institute's ethical standards and the 1964 Helsinki Declaration and its later amendments. In addition, this study was reviewed and approved by the Ethics Committee of Changhai Hospital affiliated to Naval Medical University (No. 2020-54), and participants gave consent to complete the online survey.

### 2.2. Participants

The study was conducted amongst Chinese NBDs who delivered burn care from March to June 2020. Eligible participants were nurses who: (a) were registered nurses; (b) had work experience in burn departments (burn wards or BICUs) over one year; (c) gave informed consent and voluntary participation in this study. NBDs absent from work or taking time off for illness throughout the survey period were excluded.

### 2.3. Data Collection

We calculated the sample size of this study using the following formula: *N* = *Z*^2^ *∗* *S*^2^/*d*^2^, with *Z* (95% confidence level) = 1.96, *S* (overall standard deviation) = 0.8, and *d* (permissible error) = 10%. The minimum sample size *N* was obtained as 246, and we decided to deliver 296, considering a nonresponse rate of 20%. Through Wen Juanxing (https://www.wjx.cn, an online data collection website), we distributed an anonymous online survey (https://www.wjx.cn). We trained the administrators in charge of the participants over the phone and with WeChat to help survey before the questionnaires were distributed. Sampling was separated for each institution. All the participants responded to the online Wen Juanxing survey independently by scanning the QR Code via WeChat without the presence of researchers or nursing administrators. With a response rate of 98.6%, a total of 292 NBDs working in burn departments at 12 tertiary institutions were recruited. There were no missing items in the 292 completed surveys because of the restriction of the answer system settings, but 25 of them were invalid and were removed from the dataset because of the detected all-the-same options. As a result, we received 267 valid questionnaires, and the effective response rate was 90.2%.

### 2.4. Measures

We collected data via online questionnaires, which included a socio-demographic questionnaire and the NBD Core Competencies Self-rating Scale (NBD-CCSS). The socio-demographic questionnaire collected general information such as age, professional title, length of employment, whether the participant had work experience in BICUs, and educational level. Based on a systematic literature review, we previously used the core competencies in the ICN framework for nurse specialists as a theoretical framework to develop NBD-CCSS [15]. The ICN framework was chosen due to its recommended competencies of a specialist nurse, which include knowledge, skills, judgment, and attributes, all under the premise of ethical and legal compliance. Health promotion, nursing process, therapeutic communication, and interpersonal relationships are among the fundamental care principles. However, the NBD-CCSS lacked dimensions on psychosocial help, aftercare guidance, end-of-life care, and team collaboration compared to the ABA's Burn Nurse Competencies. In China, we have not yet constructed tertiary hospitals radiating to community rehabilitation and postrehabilitation psychosocial support.

The NBD-CCSS includes nine dimensions and 100 items (see supplement 1). The nine dimensions are basic specialized knowledge, related specialized knowledge, basic specialized skills, related specialized skills, condition assessment, adverse nursing events, mass casualty care, critical thinking, and teaching skill. The items were rated using a Likert scale ranging from 1 (strongly disagree) to 5 (strongly agree). Sixteen experts assessed the content validity of the measure. The NBD-CCSS items' content validity index (I-CVI) was 0.8–1, while the scale's overall content validity index (S-CVI) was 0.976. The internal consistency test revealed that the NBD-alpha CCSS's Cronbach's coefficient was 0.984 and that each dimension's Cronbach's coefficient ranged from 0.824 to 0.958. The scale underwent exploratory factor analysis, and the entire scale's KMO coefficient was 0.951.

### 2.5. Statistical Analysis

SPSS (version 25.0) and Mplus (version 8.4) were used for data analysis. All the missing data were not included in the statistical analysis because 4 questionnaires were not submitted and 25 questionnaires had identical answers. After the data were cleaned, descriptive statistics were conducted for all variables. Continuous variables are displayed in mean ± standard deviation, and categorical variables were displayed in frequency and percentage. To accomplish the study aims, we identified latent profiles (subgroups) of NBDs' core competencies based on the nine dimensions of NBD-CCSS. Based on a specific set of factors, LPA enables the identification of latent subgroups within the population. To start, we determined the number of subgroups using the Bayesian information criterion (BIC), entropy, and bootstrapped likelihood ratio test (BLRT). Lower values of the BIC indicate higher fitness as it takes into account model fit and parsimony. Individual classification accuracy is referred to as entropy (values close to 1 are preferred). Significant *p* values indicate that the *k*-class model has more excellent fitness when BLRT compares it to the *k* − 1 class model.

Meanwhile, the one-way analysis of variance (ANOVA) and Chi-square analysis were used to analyze the distribution of the identified classes and their relationships with socio-demographic variables. Furthermore, we performed multinomial logistic regression to investigate the variables influencing profile membership. A statistically significant difference was accepted at a two-sided*p*-value <0.05.

## 3. Results

### 3.1. Fit Statistics for Latent Profiles

In this study, LPA was performed on NBDs' core competencies. We assumed equal variance between but zero covariance profiles while estimating for 2–5 profile models. The results of LPA are shown in Table 1. When comparing models, if the AIC and BIC are smaller, the entropy is higher, and the BLRT-p is less than 0.05, and the better the model fit is. As can be seen in Table 1, the 3-profile model has the highest entropy. The 4-profile model was not significantly better than the 3-profile model (*p*-value close to the critical value of 0.05) and had the lowest entropy. The 5-profile model had the lowest ABIC and AIC, but the proportion for the most minor class was only 0.022. Therefore, combining all indicators and model simplicity, the 3-profile model was found to be the optimal model for interpretation and additional analysis.

### 3.2. Distribution of Core Competencies in the 3-Profile Model

**Figure 1:**
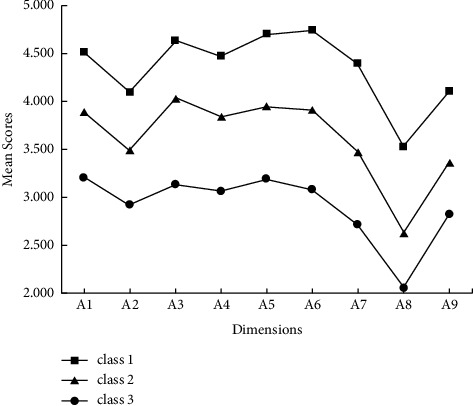
Distribution of core competencies in the 3-profile model.

The distribution of core competencies in the 3-profile model is described in Table 2 and Figure 1. The mean scores of the NBD-CCSS's dimensions significantly differed among the 3 profiles (all *p* < 0.001). Class 1, the second largest profile (*n* = 77, 28.8%), had the highest levels of core competencies. Class 2 was the largest group (*n* = 140, 52.4%), while class 3 formed the smallest group (*n* = 50, 18.7%) and was characterized by the lowest levels of core competencies. Based on the results, class 1 was labeled as “skillful competencies,” class 2 was labeled as “moderate competencies,” and class 3 was labeled as “poor competencies.”

### 3.3. Interprofile Characteristic Differences

The differences among the profiles concerning socio-demographic variables were examined (Table 3). Among the socio-demographic variables, significant differences were noted across profiles in age, professional title, education, length of employment, and experience in BICU (all *p* < 0.05). In particular, Class 3 included the largest proportion of NBDs who were ≤25 years old, junior nurses, had junior college degrees, had less than three years of employment, and had no BICU experience.

### 3.4. Variables Associated with the Latent Profile Membership

In order to analyze whether different socio-demographic variables lead to different profiles of core competencies in NBDs, a multinomial logistic regression analysis was conducted. In this study, profile membership (Class 1–Class 3) was used as the outcome variable, and the predictor variables were age, professional title, length of employment, experience in BICU, and education. During the analysis, Class 3 was set as the reference group. The final results are shown in Table 4.

It can be seen that professional title, length of employment, and experience in BICU impacted profile membership, while age and education had no significant impact. In the comparison between Class 1 and Class 3, senior nurses were more likely to be grouped in Class 3 compared to supervisor (or above) nurses. However, in the comparison between Class 2 and Class 3, there was no significant tendency to profile membership for NBDs with different professional titles. Secondly, NBDs with 4-5 years of work experience were more likely to be assigned to Class 3 than those with more than 7 years. However, there was no tendency to profile membership when comparing Class 2 and Class 3 concerning the length of employment. In terms of experience in BICU, NBDs with BICU experience tended to be more likely to fall into Class 1 and Class 2, with a 2.564-fold and 1.304-fold increase in the probability of profile membership, respectively.

## 4. Discussion

This study identified three potential profiles of core competencies for NBDs: “skillful competency” at 28.8% (*n* = 77), “moderate competency” at 52.4% (*n* = 140), and “poor competency” with a percentage of 18.7% (*n* = 50). With an overall mean score of (3.69 ± 0.79), the mean core competencies of the 267 NBDs were at a medium level. Professional titles, years of employment, and BICU experience were determined as influencing factors of NBDs' profile membership of core competencies. Moreover, NBDs who were senior nurses with 4-5 years of employment were more likely to be grouped in Class 3, while those who were supervisor nurses or above, with more than 7 years of employment and BICU experience, tended to fall into Class 1.

Among the nine dimensions, the critical thinking (2.78 ± 0.88) and teaching skills (3.47 ± 0.76) dimensions were of the lowest scores—the same cases in the NBDs' three potential profiles. Yue et al's team [25] also identified low evidence-based competencies in their previous study on burn specialist nurses in Hunan Province. Additionally, for the training of specialist nurses in China, there are training programs organized by nursing associations, such as the training of intensive care specialist nurses and operating room specialist nurses. Nevertheless, such programs are desired to be more systematic and standardized since 62.7% of them do not carry out recertification (Ding et al. [26]). Most Chinese specialist nurses gain progress through experience in relevant departments instead of systematic training [27, 28, 29]; Wang et al. This could explain our findings why NBDs lack the competencies necessary to recognize clinical issues, use scientific approaches to solve problems, raise awareness of research and teaching, and integrate theory with practice. In the United States, colleges, boards, or organizations administer specialty certification programs that evaluate whether a nurse meets the criteria they have established in a specialty field of practice. Previous studies have widely reported correlations between specialty nurse certification and better patient outcomes and care quality [30]. Therefore, it should be a key concern for nursing administrators to develop a training and certification system for NBDs underlining critical thinking and teaching skills.

In addition, we also found that NBDs' socio-demographic characteristics differed across the potential profiles. NBDs' professional titles, length of employment, and BICU work experience influenced their profile membership of core competencies. NBDs who were supervisor nurses or above, had over seven years of employment and BICU experience, had more probability of being grouped into the “skillful competencies” class than those who were senior nurses, and had 3–5 years of employment and no BICU experience. However, these factors did not impact the profile membership of the “moderate competencies” class. We are inclined to propose the following reasons: Firstly, there is a significant difference in the severity of burn patients, especially those with severe burns who are in critical condition and require a high level of competence in specialist burn care [31]. Therefore, their burn care requires more skilled nurses. Moreover, nurses with more seniority and BICU experience have more opportunities to care for severe burn patients. As a result, they are more likely to possess expertise in burn care, such as monitoring circulatory, respiratory, urinary, and other systems. Secondly, experienced nurses might realize their value in the care process, especially after solving complex clinical problems. This serves as their intrinsic driving force, coupled with support and empowerment from nursing administrators, which also enhances their extrinsic drive. All these drives could be facilitators for the self-transcendence of specialist nurses, as formerly reported by Wang et al. [32].

Furthermore, NBDs in the “poor competencies” profile were featured as having the largest proportion of those who were ≤25 years old (*n* = 24, 48.00%), junior nurses (*n* = 26, 52.00%), with a junior college education background (*n* = 29, 58.00%), with less than three years of employment (*n* = 22, 44.00%), and no BICU experience (*n* = 39, 78.00%). In accordance with Rizany et al's study [6], our findings gave more evidence to highlight the systematic training of novice nurses with lower educational levels. Through literature review, nursing associations have developed the concept of continuing competence, which has drawn attention worldwide [33]. Prior studies revealed that NBDs should possess emergency response capabilities due to burns' abrupt and batch nature Feng et al. [34]. Similarly, the literature on emergency nurses has also highlighted the need to understand their lack of knowledge and capability for a long-term career [35]. Hence, in addition to entry-level competencies, NBDs need to be continuously assessed regarding their core competencies. However, administrators are challenged with a lack of unified standards, practices, and frameworks for continuously assessing NBDs' competencies. This, therefore, makes our research more meaningful.

### 4.1. Recommendations for Future Research

Based on our study, we offer the following recommendations for cultivating NBDs' core competencies. It is important to provide enhanced training and support for both nursing managers and staff, as proposed by Avery and Cleaver [36]. Further research is needed to fully understand the impact of competence management methods on nurses. For example, previous studies have explored how contextualized simulation, such as burns suite [37] or Advanced Burn Life Support (ABLS) course [38], affects effective learning. Additionally, it is crucial to examine how nurses who fulfill their responsibilities can support clinical leaders. A noteworthy study conducted in Canada by Kandakoglu's team developed a system to assist administrators of nephrology departments by creating daily visit schedules and routes for nurses providing home dialysis treatment [39]. These recommendations can guide future research and help inform the development of targeted training programs to promote NBDs' core competencies.

### 4.2. Clinical Implications for Nursing Managers and Policymakers

The results of this study hold significant implications for nursing managers and policymakers who are responsible for designing and implementing training programs to recruit competent NBDs. The findings demonstrate that the professional titles, length of employment, and BICU experience of NBDs are closely linked to their core competencies. Hence, it is crucial for nurse managers to consider these subgroup characteristics when tailoring advanced practice programs for NBDs. This will enable them to match subgroups to appropriate training courses, thereby enhancing the overall quality of care provided by NBDs in hospitals. Policymakers can also utilize these study results to formulate evidence-based policy decisions related to the recruitment and retention of qualified NBDs in healthcare facilities. Overall, this study underscores the need for nursing managers and policymakers to be cognizant of the importance of targeted training programs that align with NBDs' specific core competencies.

### 4.3. Limitations of the Study

Although we made great efforts to ensure the accuracy of our data, this study still has several limitations. First, because the participants in this study were not randomly selected and primarily recruited from tertiary hospitals, the sample may not be sufficiently representative. Therefore, we advise broadening the study's scope to include more institutions. In addition, the competency assessment used self-reports, which might have led to possible bias. Several of the LPA's weaknesses should also be taken into account. On the one hand, while grouping based on LPA makes data presentation and interpretation easier, participants do not actually belong to a single group. Every participant's profile is assigned in light of the highest likelihood of belonging to a latent profile. On the other hand, although LPA makes it easier to identify associations between socio-demographic variables that may vary between profiles, it does not accurately reveal the specific factors driving these associations.

## 5. Conclusions

Based on potential profile analysis, this cross-sectional study explored the subgroup characteristics and influencing factors of NBDs' core competencies. We identified three profiles of NBDs' core competencies across the nine dimensions of NBD-CCSS, consisting of skillful competencies (Class 1, 28.8%), moderate competencies” (Class 2, 52.4%), and poor competencies (Class 3, 18.7%). NBDs in Class 1 had the best performance across all dimensions, while those in Class 3 had the reverse performance. Moreover, we found that potential influencing factors of profile membership included professional titles, length of employment, and BICU experience. Nursing administrators and educators can form alternative professional training and career development plans depending on the subgroup characteristics of NBDs' core competencies. Meanwhile, it is advised that more research be done in order to create specific training programs geared to those subgroups.

## Figures and Tables

**Table 1:** Fit statistics for 2–5 latent profile models (*n* = 267).

Model	AIC	BIC	ABIC	Entropy	LMR-p	BLRT-p	Proportion
2-Profile	3896.117	3996.559	3907.783	0.909	0.0002	0.0000	0.438/0.562
3-Profile	3443.528	3579.844	3459.361	0.937	0.0006	0.0000	0.187/0.524/0.288
4-Profile	3273.952	3446.140	3293.952	0.900	0.0471	0.0495	0.131/0.288/0.213/0.367
5-Profile	3181.985	3390.046	3206.151	0.905	0.0136	0.0000	0.022/0.139/0.281/0.210/0.348

*Note.* AIC, akaike information criteria; BIC, bayesian information criteria; ABIC, adjusted bayesian information criteria; LMR-p, lo-mendell-rubin; BLRT-p, bootstrapped likelihood ratio test.

**Table 2:** Distribution of core competencies in the 3-profile model (*N* = 267), mean (SD).

	Dimensions	Total sample (*n* = 267)	Class 1 (*n* = 77)	Class 2 (*n* = 140)	Class 3 (*n* = 50)	*F*	*p*
A1	Basic specialized knowledge	3.94 (0.57)	4.51 (0.35)	3.89 (0.33)	3.20 (0.45)	204.88	<0.001
A2	Related specialized knowledge	3.55 (0.55)	4.10 (0.32)	3.48 (0.35)	2.91 (0.37)	157.36	<0.001
A3	Basic specialized skills	4.03 (0.64)	4.63 (0.32)	4.03 (0.42)	3.12 (0.43)	223.59	<0.001
A4	Related specialized skills	3.87 (0.60)	4.46 (0.35)	3.84 (0.37)	3.05 (0.34)	233.45	<0.001
A5	Condition assessment	4.02 (0.61)	4.70 (0.24)	3.95(0.35)	3.17 (0.37)	338.68	<0.001
A6	Adverse nursing events	3.99 (0.72)	4.74 (0.36)	3.90 (0.47)	3.07 (0.44)	227.62	<0.001
A7	Mass casualty care	3.60(0.78)	4.40 (0.47)	3.47 (0.51)	2.72 (0.62)	165.23	<0.001
A8	Critical thinking	2.78 (0.88)	3.55 (0.74)	2.60 (0.69)	2.06 (0.70)	76.09	<0.001
A9	Teaching skills	3.47 (0.76)	4.11 (0.59)	3.36 (0.64)	2.83 (0.54)	72.12	<0.001
Total		3.69 (0.79)	4.36 (0.58)	3.65 (0.64)	2.90 (0.59)		

**Table 3:** Interprofile characteristic differences (*N* = 267), *N* (%).

Variables	Total sample (*n* = 267)	Class 1 (*n* = 77)	Class 2 (*n* = 140)	Class 3 (*n* = 50)	*χ* ^2^	*p*
Age					31.81	<0.001
≤25 y	63 (23.60)	7 (9.09)	32 (22.86)	24 (48.00)		
26∼30 y	86 (32.21)	23 (29.87)	47 (33.57)	16 (32.00)		
31∼35 y	57 (21.35)	21 (27.27)	31 (22.14)	5 (10.00)		
≥36 y	61 (22.84)	26 (33.77)	30 (21.43)	5 (10.00)		
Professional title					32.20	<0.001
Junior nurse	85 (31.84)	15 (19.48)	44 (31.43)	26 (52.00)		
Senior nurse	129 (48.31)	33 (42.86)	73 (52.14)	23 (46.00)		
Supervisor nurse or above	53 (19.85)	29 (37.66)	23 (16.43)	1 (2.00)		
Education					15.65	<0.001
Junior college	97 (36.70)	18 (23.38)	51 (36.43)	29 (58.00)		
Bachelor's degree or above	169 (63.30)	59 (76.62)	89 (63.57)	21 (42.00)		
Length of employment					37.88	<0.001
≤3 y	62 (23.22)	7 (9.09)	33 (23.57)	22 (44.00)		
4∼5 y	47 (17.60)	7 (9.09)	26 (18.57)	14 (28.00)		
6∼7 y	39 (14.61)	14 (18.18)	20 (14.29)	5 (10.00)		
>7 y	119 (44.57)	49 (63.64)	61 (43.57)	9 (18.00)		
Experience in BICU					7.96	0.019^*∗∗*^
Yes	99 (37.08)	36 (46.75)	52 (37.14)	11 (22.00)		
No	168 (62.92)	41 (53.25)	88 (62.86)	39 (78.00)		

**Table 4:** Multinomial logistic regression analysis of variables associated with the latent profile membership (*N* = 267).

Variables	Class 1		Class 2	
	*b*	OR (95% CI)	*b*	OR (95% CI)
Age (ref: ≥36 y)				
≤25 y	0.217	1.242 (0.063, 24.685)	−0.164	0.848 (0.068, 10.552)
26–30 y	1.014	2.756 (0.236, 32.223)	0.459	1.582 (0.172, 14.580)
31–35 y	0.142	1.152 (0.240, 5.529)	0.195	1.216 (0.275, 5.381)
Professional title (ref: supervisor nurse or above)				
Junior nurse	−2.464	0.085 (0.006, 1.143)	−1.595	0.203 (0.017, 2.368)
Senior nurse	−2.497^*∗*^	0.082 (0.009, 0.759)	−1.561	0.210 (0.024, 1.868)
Education				
Junior college (ref: bachelor's degree or above)	−0.213	0.808 (0.272, 2.404)	−0.239	0.787 (0.322, 1.928)
Length of employment (ref: >7 y)				
≤3 y	−2.154	0.116 (0.008, 1.741)	−0.807	0.446 (0.046, 4.308)
4-5 y	−2.402^*∗*^	0.091 (0.009, 0.906)	−1.170	0.310 (0.042, 2.270)
6-7 y	−0.591	0.554 (0.057, 5.372)	−0.411	0.663 (0.085, 5.163)
Experience in BICU				
Yes (ref: no)	1.271^*∗∗*^	3.564 (1.423, 8.925)	0.834^*∗*^	2.304 (1.024, 5.181)

*Note.* ^*∗*^means *p* < 0.05; ^*∗∗*^means *p* < 0.01.

## Data Availability

Data presented in this study are available on request from the corresponding author.

## Additional Points

Im*plications for Nursing Management.* Our results indicate a critical need to improve Chinese NBDs' core competencies and optimize the training system. Interventions at the departmental and organizational levels are desired to support specialized training for NBDs.

## Supplementary Materials

Supplementary Materials Nurses of burn department (NBDs) competences.
