# Supplementary material for: Current Status of Core Competencies of Chinese Nurses in Burn Departments: A Latent Profile Analysis
Source: J Nurs Manag. 2023 Apr 30;2023:8839286. doi: 10.1155/2023/8839286 (PMC11918496; doi:10.1155/2023/8839286)
Supplement: Supplementary Materials — Nurses of burn department (NBDs) competences. [file 8839286.f1.docx]

| **Nurses of Burn Departments (NBDs) Competences** | |
| --- | --- |
| **Dimensions** | **Items** |
| Basic specialized knowledge | Zones of burn |
|  | Depth of burn |
|  | Extent of burn |
|  | Staging of burn |
|  | Inhalation injury |
|  | Chemical burn |
|  | Electrical burn |
|  | Blast injury |
|  | Pediatric burn |
|  | Geriatric burn |
|  | Eye burn |
|  | Burn Shock |
|  | Burn Index |
|  | Rehydration Formula |
|  | Perioperative care of cutting scab |
|  | Perioperative Care of implant |
|  | Multiple trauma |
|  | Avulsion Injuries |
|  | Pressure Injuries |
|  | Diabetic foot |
|  | ARDS |
| Related specialized knowledge | Fluid types |
|  | Antibiotics |
|  | Dressings |
|  | Ttopical drugs and antiseptics |
|  | Vasoactive drugs |
|  | Analgesics |
|  | Sedatives |
|  | Narcotics |
|  | ICU syndrome |
|  | Shock index |
|  | Oxygenation index |
|  | Hemodynamic indices |
|  | Laboratory test indicators |
|  | Critical Care Rehabilitation |
|  | Nosocomial infection control |
|  | Wound management |
| Basic specialized skills | Turning bed |
|  | Suspension bed |
|  | Pediatric burn bed |
|  | Burn position |
|  | Facial care |
|  | Eye irrigation |
|  | Burn wound hydrotherapy |
|  | Arterial puncture placement |
|  | Cardiopulmonary resuscitation |
| Related specialized skills | Suctioning |
|  | Airway humidification |
|  | Nebulization |
|  | CRRT |
|  | Microvenous cannulation |
|  | Ventilator use |
|  | Use of monitor |
|  | Use of vibrating sputum meter |
|  | Measurement of urine PH |
|  | Measurement of gastric PH |
|  | Facial organ drip |
|  | Fundamental nursing |
|  | Central venous pressure |
|  | Measurement of blood glucose |
|  | High-flow nasal cannula/manual airway oxygen therapy |
|  | Nasal jejunal tube maintenance |
|  | Standardized use of oropharyngeal airway |
|  | Standardized use of nasopharyngeal airway |
|  | Central venous cannulation coordination |
|  | Fibroscopy care coordination |
|  | Ventilation in prone position |
|  | Enteral and parenteral nutrition |
|  | Maintenance of PICC |
|  | Defibrillator |
|  | Critical care transport |
|  | Bedside crustectomy care coordination |
| Condition assessment | Assessment vital signs and oximetry |
|  | Consciousness, pupils |
|  | Assessment, medication, and observation of pain |
|  | Assessment and observation of urine output |
|  | Assessment and judgment of patient complaints |
|  | Assessment and judgment of chest signs |
|  | Assessment and judgment of abdominal signs |
|  | Assessment and judgment of mental and emotional status |
|  | Assessment of sleep |
|  | Assessment of defecation |
|  | Delirium |
|  | Assessment, judgment and management of common complications |
| Adverse nursing events | Artificial airway events |
|  | Drainage tubes events |
|  | Medication events |
|  | Unintentional injuries |
| Mass casualty care | Nursing personnel assessment |
|  | Assessment of supplies |
|  | Environment preparation |
|  | Organizational and management skills |
|  | Communication skills |
|  | Emergency surgery coordination |
| Critical thinking | Literature review |
|  | Identification of clinical problems |
|  | Research Awareness |
| Teaching skills | Teaching Awareness |
|  | Knowledge acquisition |
|  | Integration of theory with practice |
